# Estimated Doses to the Heart, Lungs and Oesophagus and Risks From Typical UK Radiotherapy for Early Breast Cancer During 2015–2023

**DOI:** 10.1016/j.clon.2024.05.002

**Published:** 2024-09

**Authors:** F. Holt, A. Ivanova, Z. Wang, S. Darby, F. Duane, G. Ntentas, S. Oliveros, B. Lavery, K. Shah, A. Eichholz, D. Dodwell, C. Taylor

**Affiliations:** ∗ Nuffield Department of Population Health, University of Oxford, UK; † Canadian Nuclear Safety Commission, Ottawa, Canada; ‡ St. Luke's Radiation Oncology Network and Trinity St. James' Cancer Institute, Ireland; § Department of Medical Physics, Guy's & St Thomas' NHS Foundation Trust, UK; ¶ Oxford University Hospitals, Oxford, UK; || Buckinghamshire Healthcare NHS Trust, Aylesbury, UK

**Keywords:** Breast cancer radiotherapy, Heart dose, Lung dose, Oesophagus dose, Radiotherapy risks, UK radiotherapy

## Abstract

**Purpose:**

Breast cancer radiotherapy can increase the risks of heart disease, lung cancer and oesophageal cancer. At present, the best dosimetric predictors of these risks are mean doses to the whole heart, lungs and oesophagus, respectively. We aimed to estimate typical doses to these organs and resulting risks from UK breast cancer radiotherapy.

**Methods:**

A systematic review and meta-analysis was conducted of planned or delivered mean doses to the whole heart, lungs or oesophagus from UK breast cancer radiotherapy in studies published during 2015–2023. Average mean doses were summarised for combinations of laterality and clinical targets. Heart disease and lung cancer mortality risks were then estimated using established models.

**Results:**

For whole heart, thirteen studies reported 2893 doses. Average mean doses were higher in left than in right-sided radiotherapy and increased with extent of clinical targets. For left-sided radiotherapy, average mean heart doses were: 2.0 Gy (range 1.2–8.0 Gy) breast/chest wall, 2.7 Gy (range 0.6–5.6 Gy) breast/chest wall with either axilla or supraclavicular nodes and 2.9 Gy (range 1.3–4.7 Gy) breast/chest wall with nodes including internal mammary. For right-sided radiotherapy, average mean heart doses were: 1.0 Gy (range 0.3–1.0 Gy) breast/chest wall and 1.2 Gy (range 1.0–1.4 Gy) breast/chest wall with either axilla or supraclavicular nodes. There were no whole heart dose estimates from right internal mammary radiotherapy. For whole lung, six studies reported 2230 doses. Average mean lung doses increased with extent of targets irradiated: 2.6 Gy (range 1.4–3.0 Gy) breast/chest wall, 3.0 Gy (range 0.9–5.1 Gy) breast/chest wall with either axilla or supraclavicular nodes and 7.1 Gy (range 6.7–10.0 Gy) breast/chest wall with nodes including internal mammary. For whole oesophagus, two studies reported 76 doses. Average mean oesophagus doses increased with extent of targets irradiated: 1.4 Gy (range 1.0–2.0 Gy) breast/chest wall with either axilla or supraclavicular nodes and 5.8 Gy (range 1.9–10.0 Gy) breast/chest wall with nodes including internal mammary.

**Conclusions:**

The typical doses to these organs may be combined with dose-response relationships to estimate radiation risks. Estimated 30-year absolute lung cancer mortality risks from modern UK breast cancer radiotherapy for patients irradiated when aged 50 years were 2–6% for long-term continuing smokers, and <1% for non-smokers. Estimated 30-year mortality risks for heart disease were <1%.

## Highlights

• Average mean whole organ doses were ∼2 Gy for heart, 3 Gy lung, and 4 Gy oesophagus.
• Organ doses increased with increasing targets irradiated.
• Estimated absolute 30-year radiation heart disease mortality risks were 0.2%–0.7%.
• Equivalent lung cancer risks were 0.2%–0.4% for non-smokers and 2.3%–6.2% for smokers.

## Introduction

Adjuvant radiotherapy is received by around two-thirds of patients as part of their treatment for early breast cancer in the UK [1]. Radiotherapy reduces the risk of breast cancer death when targeted at the whole breast after breast conserving surgery or, in lymph node positive breast cancer, when targeted at the chest wall after mastectomy, with additional survival benefits when targeted also at the regional lymph nodes [[2], [3], [4]]. Randomised trials have, however, shown that radiotherapy can increase the risks of death from heart disease, lung cancer and oesophageal cancer [5].

To inform clinical decisions, oncologists need to weigh the absolute magnitudes of the benefits from radiotherapy against the absolute magnitudes of its risks. Radiation risks of heart disease, lung cancer and oesophageal cancer vary according to the doses received by the heart, lungs and oesophagus respectively. Organ doses in breast cancer radiotherapy vary according to individual anatomy, breast cancer laterality, radiotherapy targets irradiated and techniques used [[6], [7], [8]]. For a given combination of technique and target, organ doses may also vary according to geographical region. This is because techniques may be applied differently across different cancer centres. In previous systematic reviews, heart and lung doses from tangential breast or chest wall radiotherapy were typically lower in the UK than in some other countries [7,8]. This may be due to availability of specialised techniques [[9], [10], [11], [12], [13], [14], [15], [16]].

In this study, we estimate typical mean doses to the whole heart, lungs and oesophagus from modern UK breast cancer radiotherapy and the extent to which they vary. We focus on mean organ doses because the dose-response relationships with the largest numbers of events in patients with individual dosimetry, are based on mean doses to the heart, lungs and oesophagus [17]. We then illustrate how these typical mean doses might be used to estimate the absolute risks of death from heart disease and lung cancer from UK radiotherapy by combining them with published dose-response relationships and cause-specific mortality rates.

## Methods

A systematic review of mean radiation doses to the whole heart, lungs or oesophagus from UK radiotherapy for early breast cancer published during 2015–2023 was performed with reference to Preferred Reporting Items for Systematic Reviews and Meta-Analyses guidelines [18]. This date range was used because our aim was to assess modern radiotherapy. The review was registered on the International Prospective Register of Systematic Reviews [19].

### Study Eligibility

Eligible studies were those reporting planned or delivered mean whole organ doses (Gy) to the heart, lungs or oesophagus from UK radiotherapy for early breast cancer published between 1st January 2015 and 12th June 2023. Eligible studies included at least one radiotherapy plan generated since 1st January 2015. Studies where only some dose estimates were produced during 1st January 2015 to 12th June 2023 were included. A similar approach was taken for radiotherapy that had been re-planned as part of a study, regardless of whether dates were specified, if the publication date fell within 1st January 2015 to 12th June 2023. We excluded studies that specified all radiotherapy was planned prior to 2015.

Studies were categorised as dosimetry (i.e., studies that reported doses from radiotherapy plans done for research purposes only), observational/audit (i.e., studies that reported doses delivered to patients in routine practice only), dosimetry & observational (i.e., studies that included doses in both research and routine practice) and interventional (i.e., studies that reported doses received by patients in clinical trials). Early breast cancer was defined as disease confined to the breast or axillary lymph nodes that could be removed surgically. Patients of any age or sex were included. For the heart and oesophagus, whole organ doses were eligible. For the lungs, whole organ dose referred to the dose distributed among both lungs. Studies that reported separate mean ipsilateral lung dose and mean contralateral lung dose were eligible since these doses could be averaged to estimate mean whole lung dose.

### Study Identification

Eligible studies were identified by searching MEDLINE and Embase. The search strategy used terms for breast cancer, radiotherapy and dose (Text S1). Studies from the UK were identified using a National Institute for Health and Care Excellence (NICE) validated search filter which has been shown to retrieve UK-focused evidence with high recall [20]. Two reviewers (AI and FH) independently assessed titles and abstracts returned by the search for eligibility. To minimise the risk of including duplicate study cohorts, study datasets were compared using available information on geographical location, dates of radiotherapy plans, radiotherapy descriptions and numbers of patients irradiated. Studies were excluded if the doses in them were already reported first in another publication (e.g. some reviews).

### Data Extraction

A data extraction form was designed, with definitions of treatment planning methods and radiotherapy techniques informed by previous similar reviews (Text S2) [[6], [7], [8]]. Two reviewers (AI and FH) independently extracted the data using Covidence systematic review software [21]. Differences were resolved through consensus.

From each included study the following information was sought: first author, year of publication, geographical location of the cancer centre where the radiotherapy was planned, study design and years during which radiotherapy was planned or delivered. The radiotherapy description included treatment planning methods, radiotherapy technique, beam type and energy, use of breathing control, laterality of the cancer(s), clinical targets, target dose and fractionation, and whether a boost to the tumour bed was given. Patient treatment position and contouring guidelines were also noted.

Dose terms were as follows: “whole organ dose” refers to the dose averaged over the whole organ. ‘Mean whole organ dose’ is the mean of whole organ doses for a particular group of patients in a study (i.e., each row of tables S2, S4 or S6). ‘Average mean whole organ dose’ is the average dose for several groups of patients combined (i.e., several rows in tables S2, S4 or S6). Dose statistics extracted were the mean (or median, where mean was not reported), standard deviation and range for the whole heart, lung or oesophagus. For each mean whole organ dose statistic, the total number of contributing individual dose estimates was recorded. The total number of patients and CT planning scans from which individual dose estimates were created were counted, as were the number and percentage of dose estimates delivered to patients. Some dosimetry studies may have included CT scans and/or radiotherapy plans that had been previously used in other studies, however, to the best of our knowledge the reported individual organ dose estimates were unique to each study.

The number of patients described as having unusual anatomy was sought. For one study (IMPORT HIGH trial), mean whole organ doses were reported in six categories of target dose and laterality, but the number of individual patients, and hence dose estimates, in each category was not reported [22]. The likely number of patients in each of the six categories was therefore estimated by considering the published percentages of patients who received left and right-sided radiotherapy within each of the dose levels in the trial (56Gy/53Gy/48Gy).

### Data Analysis

Included studies were grouped according to the organ(s) at risk for which they reported doses. Clinical targets were grouped as: breast or chest wall only (Br/CW only); breast or chest wall with either axilla or supraclavicular nodes (Br/CW + Ax/SF); breast or chest wall with either axilla or supraclavicular nodes, also including internal mammary nodes (Br/CW ± Ax/SF + IM). Laterality for each mean whole organ dose estimate was defined as all patients had left-sided cancer (L); all patients had right-sided cancer (R); some patients had left- and some had right-sided cancer (L/R). No patients were reported with bilateral cancer.

For heart, dose estimates were categorised first by breast cancer laterality, then by radiotherapy clinical targets. For lung, ipsilateral and contralateral lung doses were averaged to estimate whole lung dose, since the dose-response relationship for radiation-induced lung cancer is based on whole lung dose [5]. Mean whole lung doses were then categorised by radiotherapy clinical targets. For oesophagus, mean whole oesophagus doses were categorised by clinical targets.

For each combination of radiotherapy clinical targets and laterality, the overall average mean whole heart dose was calculated by weighting the mean whole heart dose in each study by the number of individual dose estimates it was based on. The standard error (SE) of each overall average was then calculated in similar fashion [23]. Tests of heterogeneity between the overall average mean whole heart dose in the three laterality groups and tests of trend in average mean whole heart dose across the extent of the clinical targets irradiated were carried out using variance-weighted least squares. Average mean whole lung and oesophagus doses and trends across the extent of clinical targets irradiated were carried out similarly. Calculations were carried out using the data analysis tool available in the statistical software R version 4.2.2 [24].

### Analyses Using Individual Patient Doses in a Single UK Centre

Organ doses received in routine practice may be higher than those in articles where authors have chosen to publish their doses. Therefore, the extent to which published laterality- and target-specific doses are reflected in routine clinical practice was assessed. Organ doses were estimated for patients irradiated in one cancer centre (Oxford). Fifty consecutive patients in the centre's CT planning database who received radiotherapy for early breast cancer during May and June 2018 were selected. The heart, lungs and oesophagus were contoured and individual patient whole organ doses were estimated. For each organ, mean whole organ doses were then estimated for different clinical situations.

## Results

Fourteen studies published during 2015–2023 reported mean whole organ doses (Gy) to the heart, lungs and/or oesophagus from UK radiotherapy for early breast cancer (Figure S1, Text S3). Heart dose was reported in 13 studies, lung dose in 6 studies and oesophagus dose in 2 studies.

### Heart Dose

**Fig 1:**
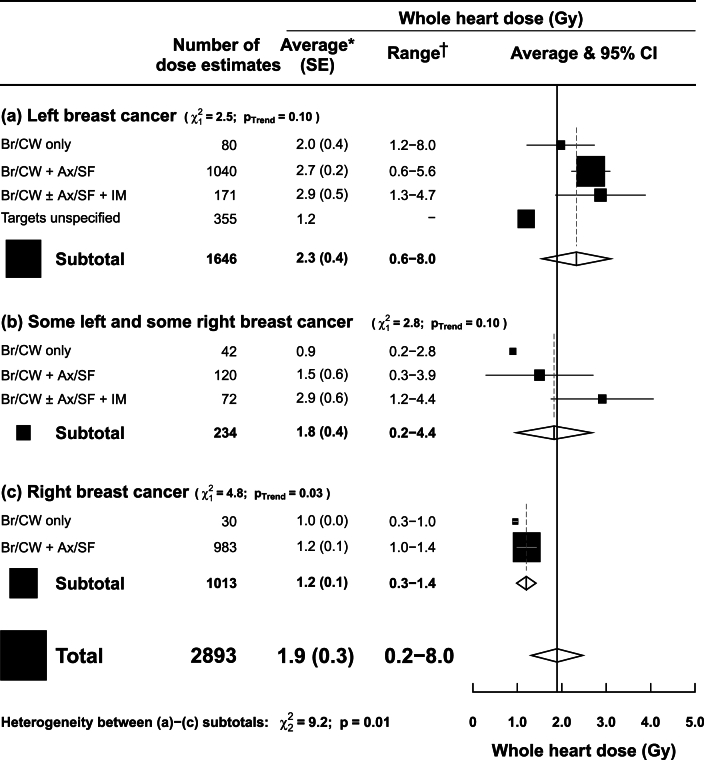
Estimated typical mean whole heart doses from UK breast cancer radiotherapy according to breast cancer laterality and clinical targets. ∗Average mean whole heart dose, weighted by the number of individual dose estimates, was calculated for each category of breast cancer laterality and clinical targets. †The range refers to the range of individual dose estimates. Where this was unavailable, the range of reported mean doses was used. P_trend_ values are for association of average dose with increasing targets irradiated. Abbreviations: Ax=axilla lymph nodes, Br=breast, CI=confidence interval, CW=chest wall, Gy=gray, IM=internal mammary lymph nodes, SE=standard error, SF=supraclavicular fossa lymph nodes.

Mean whole heart doses were reported from a total of 2893 individual dose estimates (Figure 1, Tables S1, S2). Of these, 2505 (87%) were delivered to patients. The other 388 (13%) were from dosimetry studies (Table S1). Two large studies included 2313 (80%) of the doses. The largest study reported doses in a clinical trial (IMPORT HIGH), involving 39 radiotherapy centres throughout the UK and 1958 patients [22]. Patients in IMPORT HIGH underwent breast cancer radiotherapy between 2009 and 2015. As the regimens from 2015 could not be separated from those of 2009–2014, this study was considered to fall within the scope of the review as per the predetermined inclusion criteria.

The second largest study was a national audit of breast cancer radiotherapy practice in the UK, reporting doses on 355 patients [25]. The audit presented summary heart doses in routine clinical practice, but doses were not provided separately according to clinical targets. The remaining 11 studies each included between 10 and 98 whole heart dose estimates.

There were 2314 whole heart dose estimates (80%) from intensity modulated radiotherapy (IMRT) techniques using photons (Table S2), 28 (1%) from newer IMRT techniques using protons and 196 (7%) from older photon techniques using tangential fields with fixed gantry angles. Breathing control could not be ascertained for 2545 (88%) of whole heart dose estimates. The use of deep inspiratory breath hold was specified for 212 (7%) and free breathing for 136 (5%) of estimates (Table S2).

There were 1646 whole heart doses from left-sided radiotherapy, 1013 from right-sided radiotherapy and 234 from mixed laterality (some left and some right) (Figure 1). For left-sided radiotherapy, average mean whole heart dose (Gy) from UK radiotherapy was 2.3 Gy (SE 0.4 Gy), with a wide range (0.6–8.0 Gy) for individual patients. For right-sided radiotherapy, average mean whole heart dose was 1.2 Gy (SE 0.1 Gy, range 0.3–1.4 Gy). For mixed laterality, it was 1.8 Gy (SE 0.4 Gy, range 0.2–4.4 Gy). Average mean whole heart dose increased with the extent of clinical targets irradiated. For left-sided radiotherapy, it was 2.0 Gy (SE 0.4 Gy) from breast/chest wall only, 2.7 Gy (SE 0.2 Gy) from breast/chest wall+axilla/supraclavicular nodes and 2.9 Gy (SE 0.5 Gy) from breast/chest wall±axilla/supraclavicular+internal mammary nodes. For right-sided radiotherapy, average mean whole heart dose was 1.0 Gy (SE 0.0 Gy) from breast/chest wall only and 1.2 Gy (SE 0.1 Gy) from breast/chest wall+axilla/supraclavicular nodes. There were no whole heart dose estimates from right internal mammary radiotherapy.

In IMPORT HIGH [22], mean whole heart doses from left radiotherapy were 2.4, 2.7 and 2.9 Gy, based on 316, 319 and 340 dose estimates respectively (Table S2). In other studies, mean whole heart doses in left radiotherapy varied from 0.5 to 6.4 Gy based on between 4 and 355 dose estimates. For right radiotherapy, mean whole heart doses in IMPORT HIGH were 1.0, 1.3 and 1.4 Gy, based on 316, 340 and 327 dose estimates respectively. In other studies, mean whole heart doses in right radiotherapy were 0.9 and 1.0 Gy, each based on 15 doses.

### Lung Dose

**Fig 2:**
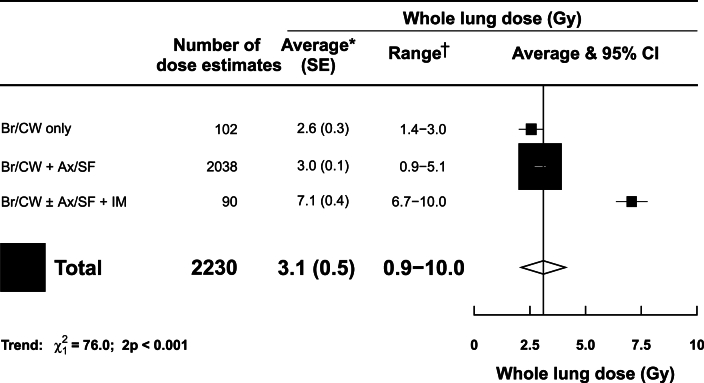
Estimated typical mean whole lung doses from UK breast cancer radiotherapy according to clinical targets. ∗Average mean whole lung dose, weighted by the number of individual dose estimates, was calculated for each category of clinical targets. †The range refers to the range of individual dose estimates. Where this was unavailable, the range of reported mean doses was used. P_trend_ values are for association of average dose with increasing targets irradiated. Abbreviations: Ax=axilla lymph nodes, Br=breast, CI=confidence interval, CW=chest wall, Gy=gray, IM=internal mammary lymph nodes, SE=standard error, SF=supraclavicular fossa lymph nodes.

Six of the fourteen studies included both ipsilateral and contralateral lung doses (Tables S3, S4). This gave rise to a total of 2230 whole lung dose estimates (Figure 2). Of these, 2049 (92%) were delivered to patients. The largest study was the IMPORT HIGH clinical trial, which included 1958 (88%) of the whole lung doses [22].

All whole lung dose estimates were from intensity modulated radiotherapy (IMRT) using photons (Table S4). Breathing control could not be ascertained for 2180 (98%) of whole lung dose estimates. The use of deep inspiratory breath hold was specified for 50 (2%) of estimates (Table S4).

There were 102 whole lung dose estimates from radiotherapy to breast/chest wall only, 2038 from radiotherapy to breast/chest wall+axilla/supraclavicular nodes and 90 from radiotherapy to breast/chest wall±axilla/supraclavicular+internal mammary nodes. Average mean whole lung dose increased with the extent of clinical targets irradiated, taking values 2.6 Gy (SE 0.3 Gy, range 1.4–3.0 Gy) from breast/chest wall only, 3.0 Gy (SE 0.1 Gy, range 0.9–5.1 Gy) from breast/chest wall+axilla/supraclavicular nodes and 7.1 Gy (SE 0.4 Gy, range 6.7–10.0 Gy) from breast/chest wall±axilla/supraclavicular+internal mammary nodes (Figure 2).

In IMPORT HIGH [22], mean whole lung doses were 2.8, 3.0 and 3.2 Gy, based on 632, 659 and 667 dose estimates respectively (Table S4). In other studies, mean whole lung doses varied from 2.0 to 8.5 Gy based on between 5 and 40 dose estimates.

### Oesophagus Dose

**Fig 3:**
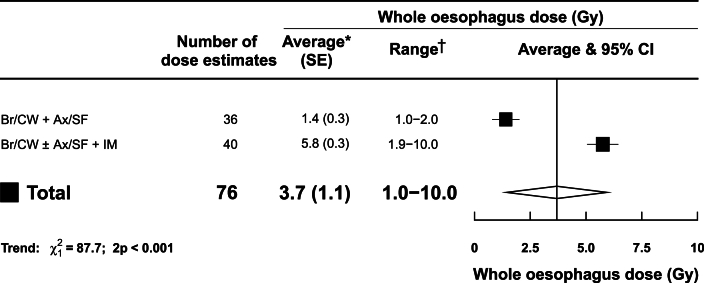
Estimated typical mean whole oesophagus doses from UK breast cancer radiotherapy according to clinical targets. ∗Average mean whole oesophagus dose, weighted by the number of individual dose estimates, was calculated for each category of clinical targets. †The range refers to the range of individual dose estimates. Where this was unavailable, the range of reported mean doses was used. P_trend_ values are for association of average dose with increasing targets irradiated. Abbreviations: Ax=axilla lymph nodes, Br=breast, CI=confidence interval, CW=chest wall, Gy=gray, IM=internal mammary lymph nodes, SE=standard error, SF=supraclavicular fossa lymph nodes.

Two of the fourteen studies reported whole oesophagus doses from a total of 76 individual estimates. Of these 76 doses, 20 (26%) were delivered to patients and the rest were estimated for research purposes (Figure 3, Tables S5, S6).

Just over half of the whole oesophagus doses were from intensity modulated radiotherapy (IMRT) techniques using photons (40/76, 53%), with the other 36 (47%) doses from older photon radiotherapy techniques using tangential fields with fixed gantry angles (Table S6). Breathing control was not specified for any patient group. Average mean whole oesophagus dose increased with the extent of clinical targets irradiated (Figure 3). It was 1.4 Gy (SE 0.3 Gy, range 1.0–2.0 Gy) from radiotherapy to breast/chest wall+axilla/supraclavicular nodes, based on 36 individual dose estimates, and 5.8 Gy (SE 0.3 Gy, range 1.9–10.0 Gy) from radiotherapy to breast/chest wall±axilla/supraclavicular+internal mammary nodes, based on 40 individual dose estimates.

### Analyses Using Individual Patient Doses in a Single UK Centre (Text S4, Table S7)

Of 50 patients, 30 received left-sided radiotherapy and 20 right-sided radiotherapy. Patients were irradiated using forward-planned IMRT, with DIBH in left radiotherapy. Breast/chest wall radiation was received by 35 patients, breast/chest wall+axilla/supraclavicular nodes by 14 patients and breast/chest wall±axilla/supraclavicular+internal mammary radiotherapy by one patient. Mean whole heart dose was higher in left than in right-sided radiotherapy; left 1.3 Gy (range 0.6–2.9 Gy) and right 0.4 Gy (range 0.2–0.8 Gy). For lung, mean whole organ dose was 3.8 Gy (range 2.0–6.8 Gy) and for oesophagus it was 0.5 Gy (range 0.1–2.4 Gy).

Comparing mean doses in the single centre audit with average mean doses in the review of UK published doses, audit doses were lower for whole heart and oesophagus: heart, audit 1.0 Gy (range 0.2–2.9 Gy) versus review 1.9 Gy (range 0.3–8.0 Gy); oesophagus, audit 0.5 Gy (range 0.1–2.4 Gy) versus review 3.7 Gy (range 1.0–10.0 Gy). For heart and oesophagus, all target and laterality-specific organ doses were also lower in the audit than in the review.

In contrast, for whole lung, mean audit doses were slightly higher than average mean doses in the review: audit 3.8 Gy (range 2.0–6.8 Gy) versus review 3.1 Gy (range 0.9–10.0 Gy). Target-specific doses were also slightly higher in the audit than in the review: breast/chest wall: audit 3.5 Gy versus review 2.6 Gy and breast or chest wall+axilla/supraclavicular fossa lymph nodes: audit 4.6 Gy versus review 3.0 Gy.

## Discussion

This systematic review and meta-analysis provides up-to-date estimates of typical doses to the whole heart, lungs and oesophagus from UK radiotherapy for early breast cancer. Published average mean whole organ doses were around 2 Gy for heart, 3 Gy for lung and 4 Gy for oesophagus. For all organs, doses increased with the extent of radiotherapy clinical targets included. Heart doses also varied according to breast cancer laterality.

### Strengths and Limitations

This study has several strengths. It summarises doses received by all three organs (heart, lung and oesophagus) implicated in the main mortality risks of breast cancer radiotherapy [5]. To the best of our knowledge it is the largest quantitative synthesis of organ doses from modern UK breast cancer radiotherapy. Studies were identified and data extracted independently by both an oncologist and a radiation health sciences officer. The literature search used a validated method of identifying UK studies and the analyses used an established method of dose meta-analysis [[6], [7], [8],^20^]. The search strategy was wide, so it is likely that all relevant studies were identified. Summary doses were based on more than 5000 individual dose estimates in fourteen studies. Average mean whole organ doses were weighted according to the number of patients contributing to each, so that the doses for different clinical scenarios are likely to be representative of typical doses received by patients in these scenarios. An audit of 50 patients in a single cancer centre enabled the published doses to be compared with doses in routine clinical practice in patients irradiated according to national UK guidance. The extent to which the doses received by these patients reflect practice in other UK centres is not known. Routine UK practice can be assessed by national audits where all cancer centres are invited to contribute data. For heart, mean whole organ doses from a national audit were included in our study, with 355 patients from 47 of the 58 UK radiotherapy centres [25]. National audit doses were unavailable for lung and oesophagus. In future, national collection of organ doses may enable more comprehensive assessment of routine practice.

There were several limitations in our study. First, individual patient doses were not usually reported in publications, so standard error and range could not be presented for some mean whole organ doses. Second, methods of contouring the organs were rarely reported for included studies. This may have contributed to the wide range of reported doses. Third, only 76 estimates of oesophagus dose were available. Fourth, dose-volume measures were not extracted. Previous reviews found that comparable heart and lung dose-volume parameters in contemporary breast cancer radiotherapy were infrequently reported [7,8]. Nevertheless, mean whole organ doses are currently the best available predictors of radiation-induced heart disease and cancer [17]. Future epidemiological studies including detailed individual patient three-dimensional doses to the heart, lungs or oesophagus may provide further information on whether dose-volume measures, or doses to parts of organs, improve prediction of radiation-induced heart disease or cancer [26,27].

A fifth limitation of our study is that most heart and lung doses were from a single clinical trial (IMPORT HIGH). Practice in radiotherapy centres that recruited to this trial may differ from routine practice in other centres. For example, centres recruiting to the trial had contouring protocols and dosimetry quality assurance, which may not be available in other centres. However, 39 of the 58 (67%) UK centres entered patients into IMPORT HIGH, so the organ doses from this trial represent practice in the majority of UK centres. In addition, the 39 centres that recruited to IMPORT HIGH are likely to treat more patients per year than the other 19 centres that did not recruit to IMPORT HIGH. Therefore the radiotherapy practices in these 39 centres are likely to reflect those available to most UK breast cancer patients. In addition, large multicentre trials such as IMPORT HIGH have improved consistency and increased quality assurance nationwide in routine radiotherapy.

### Typical Absolute Radiation-Induced Mortality Risks

The average mean whole organ doses from different combinations of breast cancer laterality and clinical targets irradiated may be used to estimate typical radiation mortality risks using published dose-response relationships (Text S5). For an individual, the absolute risk of death from heart disease or lung cancer attributable to radiotherapy may be estimated using their background death rate in the absence of radiotherapy together with the percentage increase in death rate arising from radiotherapy for the organs exposed.

For ischaemic heart disease, the mortality rate was estimated by Darby *et al.* (2013) to increase by 7.4% per Gy (95% CI 2.9–14.5) mean whole heart dose [28]. The study included 963 irradiated women with major coronary events and 1205 irradiated controls. In that study the mean radiation dose to the heart was a better predictor of the rate of major coronary events than the mean dose to the left anterior descending coronary artery. In addition, the mean dose to the left anterior descending coronary artery was not significantly associated with the rate of major coronary events after the mean dose to the heart was taken into account. In a sub-study of the 456 cases with available information on the site of cardiac injury, mean segment dose was associated with injury for all individual segments of the left ventricle and coronary arteries [29]. For estimation of cardiac radiation-risks in the clinic today, the mean whole heart dose is the best predictor currently available. However, some uncertainties remain, including the possible effects of fractionation or beam modality on the slope of the dose-response.

**Fig 4:**
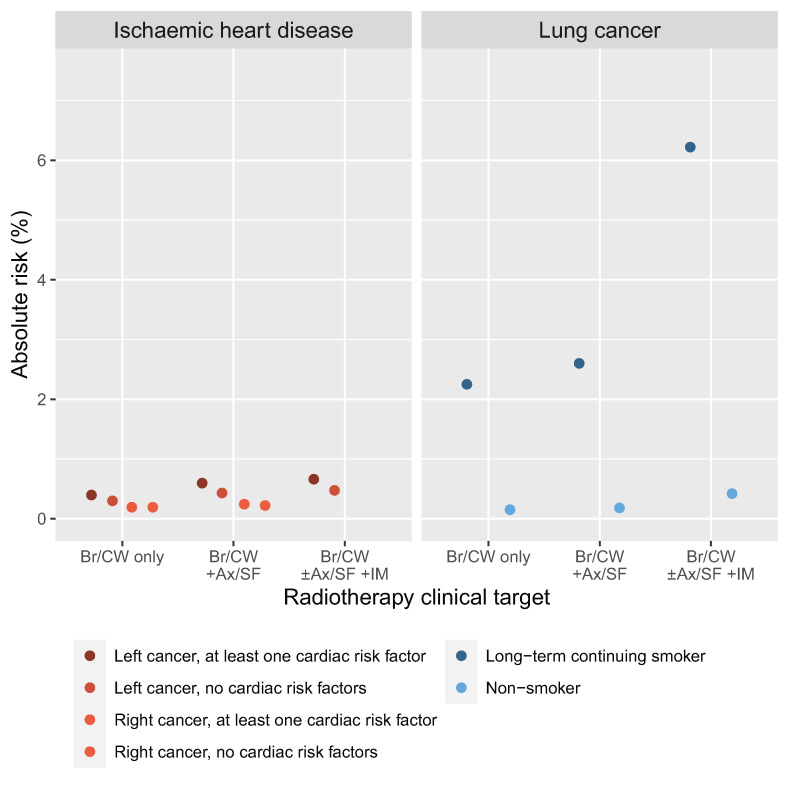
Estimated typical absolute 30-year risks (%) of radiotherapy-induced mortality for a patient who receives modern UK breast cancer radiotherapy with typical heart and lung doses when aged 50 years. Absolute risks were estimated using average mean doses to whole organs at risk (Gy) from modern UK radiotherapy for early breast cancer (Fig 1, Fig 2) and the dose-response relationships described in Text S5. Cardiac risk factors include smoking, see Text S5 for details. The risks are shown in Table 1. Risks of radiation-induced lung cancer mortality in ex-smokers are likely to be much closer to those in never-smokers than in current smokers because stopping smoking substantially reduces lung cancer risk. Figure 1 shows average mean whole heart doses for nine categories of laterality and target. Absolute risks in Table 1 and in this figure are shown for the following five categories, i.e., all categories excluding “targets unspecified” and “some left and some right”: Breast cancer laterality and target: Average mean whole heart dose (Gy), Left Br/CW only: 2.0, Left Br/CW+Ax/SF: 2.7, Left Br/CW±Ax/SF+IM: 2.9, Right Br/CW only: 1.0, Right Br/CW+Ax/SF: 1.2, Abbreviations: Ax=axilla lymph nodes, Br=breast, CW=chest wall, IM=internal mammary lymph nodes, SF=supraclavicular fossa lymph nodes.

An indication of absolute risks from typical doses in different clinical situations was given in the online Appendix of Darby 2013 [28] by combining the 7.4% per Gy dose-response with population-based mortality rates for ischaemic heart disease. Some of these absolute risks are reproduced in Text S5 and can be used to give approximate indications of the cardiac risks for patients today. The average mean heart doses found in our study with known laterality and targets varied from 1.0 to 2.9 Gy (Figure 1). This suggests that, for a patient aged 50 years when irradiated, typical UK radiotherapy would increase their absolute 30-year risk of death from ischaemic heart disease by between 0.2% and 0.7% (Table 1, Figure 4, Text S5). These estimates do not take into account the competing risk of death from breast cancer. In addition, death rates from ischaemic heart disease have reduced during the past 20 years, so the radiation-induced heart disease mortality risks for patients today may be somewhat lower than this.

**Table 1:** Estimated typical absolute 30-year risks (%) of radiotherapy-induced mortality for a patient who receives modern UK breast cancer radiotherapy with typical heart and lung doses when aged 50 years

**Targets irradiated**	**Absolute risks (%) of radiotherapy-induced mortality**					
	Ischaemic heart disease				Lung cancer	
	Left cancer, no cardiac risk factors	Left cancer, ≥1 cardiac risk factor	Right cancer, no cardiac risk factors	Right cancer, ≥1 cardiac risk factor	Non-smoker	Long-term continuing smoker
Br/CW only	0.3	0.4	0.2	0.2	0.2	2.3
Br/CW +Ax/SF	0.4	0.6	0.2	0.2	0.2	2.6
Br/CW ±Ax/SF +IM	0.5	0.7	–	–	0.4	6.2

Footnotes.

Absolute risks were estimated using average mean doses to whole organs at risk (Gy) from modern UK radiotherapy for early breast cancer (Fig 1, Fig 2) and the dose-response relationships described in Text S5. Cardiac risk factors include smoking, see Text S5 for details. These data are illustrated in Figure 4. Risks of radiation-induced lung cancer mortality in ex-smokers are likely to be much closer to those in never-smokers than in current smokers because stopping smoking substantially reduces lung cancer risk.

Figure 1 shows average mean whole heart doses for nine categories of laterality and target. Absolute risks in this table and Figure 4 are shown for the following five categories, i.e., all categories excluding “targets unspecified” and “some left and some right.”

Breast cancer laterality and target: Average mean whole heart dose (Gy).

Left Br/CW only: 2.0.

Left Br/CW+Ax/SF: 2.7.

Left Br/CW±Ax/SF+IM: 2.9.

Right Br/CW only: 1.0.

Right Br/CW+Ax/SF: 1.2.

Abbreviations: Ax=axilla lymph nodes, Br=breast, CW=chest wall, IM=internal mammary lymph nodes, SF=supraclavicular fossa lymph nodes, "–"=doses were unavailable for these categories.

For lung cancer, in an EBCTCG meta-analysis, the rate of incident radiation-induced lung cancer increased by 11% per Gy mean whole lung dose [5]. The EBCTCG publication applied this dose-response relationship to population mortality data, suggesting an absolute increase in risk of lung cancer death from 5 Gy whole lung dose of 0.3% for a non-smoker and 4.4% for a long-term continuing smoker. Whole lung doses in typical UK breast cancer radiotherapy in our study were usually lower than 5 Gy. The average mean whole lung dose for radiotherapy to the breast/chest wall was 2.6 Gy, for radiotherapy to the breast/chest wall+axilla/supraclavicular nodes it was 3.0, and for breast/chest wall±axilla/supraclavicular+internal mammary nodes it was 7.1 Gy (Figure 2). If absolute lung cancer mortality risk increased linearly with increasing dose, the corresponding 30-year absolute risks from these doses for a lifelong non-smoker would be 0.2%, 0.2% and 0.4% respectively, while the corresponding risks for a long-term continuing smoker would be 2.3%, 2.6% and 6.2% (Table 1, Figure 4, Text S5). The risks of radiation-induced lung cancer mortality in ex-smokers are likely to be much closer to those in never-smokers than in current smokers because stopping smoking substantially reduces lung cancer risk, and if a patient stops smoking at the time of radiotherapy, much of the increased risk associated with smoking could be avoided [5].

For oesophageal cancer, estimates of absolute risk from different oesophagus doses are currently unavailable. However, oesophageal cancer in the general population is uncommon, so absolute oesophageal cancer death risks are likely to be lower than the risks of death from heart disease or lung cancer [30].

### Implications for Current Clinical Practice

The typical doses and absolute risks presented in this study are reassuring for the majority of patients. The current version of the Royal College of Radiologists' breast cancer radiotherapy consent form categorises the “increased risk of heart disease in later life” and “a different cancer in the treatment area” as rare (occurring with a frequency of less than 1%) [31]. This statement aligns with the evidence we present for most patients irradiated for breast cancer, who are non-smokers or ex-smokers. For patients who are long-term continuing smokers, i.e., those who are likely to keep smoking, the risks are higher than 1% and the consent form can be annotated to reflect this. These risks are particularly relevant in clinical situations where radiotherapy has not been shown to reduce breast cancer mortality, for example after surgery for carcinoma *in situ*. These doses and risks may be used in the UK to help guide clinical decisions about radiotherapy benefits and risks for patients today. They can also inform UK national guidelines on optimal dose constraints for breast cancer radiotherapy.

### Implications for Future Research

Although the methods of estimating absolute risks of death from radiation-induced heart disease and lung cancer illustrated in this study are based on the highest-quality published evidence [17], further work is needed to facilitate their routine estimation. For example, more information is needed on how the risks vary according to age at irradiation, or the type of systemic therapy given in addition to radiation. Information is also needed on the absolute risks of oesophageal cancer from different oesophagus radiation doses. Work is underway to produce estimates that take into account the competing risks of breast cancer death and other causes of death using up-to-date population data.

## Conclusions

This study provides estimates of typical doses to organs at risk from modern UK breast cancer radiotherapy and illustrates how these may be used in combination with epidemiological data to estimate absolute radiotherapy risks. For a typical 50-year old long-term continuing smoker, the estimated 30-year absolute mortality risks of lung cancer from modern UK breast cancer radiotherapy range from 2 to 6%. However, most breast cancer patients considered for radiotherapy in the UK today are non-smokers or ex-smokers with no cardiac risk factors, and their risks of lung cancer or heart disease death are likely to be less than 1%.

## Funding and Acknowledgements

Financial support for this study was provided by the National Institute of Health and Social Care Research (NIHR) [NIHR Doctoral Fellowship (NIHR300676)] and by Cancer Research UK (C8225/A21133 and PRCRPG-Nov21∖100001). Dr Ntentas reports funding from NIHR (NIHR301261), Health Education England. The views expressed are those of the authors and not necessarily those of the NIHR or the Department of Health and Social Care or Cancer Research UK. The funders had no role in study design, the collection, analysis and interpretation of data, in the writing of the report and in the decision to submit the article for publication.

We would like to acknowledge Nia Roberts for her invaluable help with the search strategy, based on her considerable experience of high quality medical searches. We are grateful to patient advocates Mairead Mackenzie and Hilary Stobart from Independent Cancer Patients' Voice who highlighted the need for information on the risks from UK breast cancer radiotherapy and provided input to interpretation of the results.

Procedures for accessing the data for this study are available on: https://www.ndph.ox.ac.uk/about/data-access-policy.

## Author Contributions

1. Guarantor of integrity of the entire study: FH/AI.

2. Study concept and design: CT/DD/SD/FH/AI.

3. Literature research: FH/AI.

4. Clinical studies: NA.

5. Data analysis: FH/ZW.

6. Statistical analysis: ZW/SD.

7. Manuscript preparation: All authors.

8. Manuscript editing: All authors.

## Conflict of Interest

The authors declare the following financial interests/personal relationships that may be considered as potential competing interests: Zhe Wang, Georgios Ntentas, Sileida Oliveros, David Dodwell reports financial support was provided by Cancer Research UK. Francesca Holt reports financial support was provided by National Institute of Health and Social Care Research (NIHR). Georgios Ntentas reports financial support was provided by NIHR (NIHR301261), Health Education England. Carolyn Taylor reports financial support was provided by Cancer Research UK. If there are other authors, they declare that they have no known competing financial interests or personal relationships that could have appeared to influence the work reported in this article.

## Statement of Searches

The search strategy is provided in the supplementary material. It was run in EMBASE and Medline and the returned records were screened against study inclusion and exclusion criteria.

## Consent

A table in Text S5 (Supplementary material p23) is based on data produced by our team and published in the supplementary material (Table S12) of Darby SC, Ewertz M, McGale P *et al.* Risk of ischemic heart disease in women after radiotherapy for breast cancer. N Engl J Med 2013; 368(11): 987-98. According to the New England Journal of Medicine author permissions, authors are allowed to include portions of their articles in other educational articles that they write.

https://www.nejm.org/author-center/permissions (accessed 3rd Jan 2024).

Tweet: UK breast cancer radiotherapy mortality risks from lung cancer or heart disease are <1% for non-smokers but may be a few percent for smokers.
