# Supplementary material for: Estimated Doses to the Heart, Lungs and Oesophagus and Risks From Typical UK Radiotherapy for Early Breast Cancer During 2015–2023
Source: Clin Oncol (R Coll Radiol). 2024 Sep;36(9):e322–32. doi: 10.1016/j.clon.2024.05.002 (PMC11511668; doi:10.1016/j.clon.2024.05.002)
Supplement: Multimedia component 1 [file mmc1.pdf]

Estimated doses to the heart, lungs and oesophagus and risks from typical UK radiotherapy for early breast cancer during 2015-2023

Supplementary material

Contents

		Page
Text S1	Literature searches	3-6
Text S2	Definitions used for data extraction	7
Figure S1	Process of study identification	8
Text S3	References for included studies	9,10
Table S1	Studies reporting whole heart doses from radiotherapy for early breast cancer published 2015-2023	11
Table S2	Mean whole heart doses (Gy) and radiotherapy descriptions from thirteen UK breast cancer radiotherapy studies published during 2015-2023	12,13
Table S3	Studies reporting ipsilateral and contralateral lung doses from radiotherapy for early breast cancer published during 2015-2023	14
Table S4	Estimated mean whole lung doses (Gy) and radiotherapy descriptions from six UK breast cancer radiotherapy studies published during 2015-2023	15
Table S5	Studies reporting whole oesophagus doses from radiotherapy for early breast cancer published during 2015-2023	16
Table S6	Mean whole oesophagus doses (Gy) and radiotherapy descriptions from two UK breast cancer radiotherapy studies published during 2015-2023	17
Text S4	Estimated radiation doses to heart, lungs and oesophagus from breast cancer radiotherapy in the Oxford Cancer Centre, 2018	18-20
Table S7	Mean whole organ doses from 50 women irradiated for early breast cancer at the Oxford Cancer Centre during 2018 compared with doses from UK breast cancer radiotherapy studies published during 2015-2023	21
Text S5	Estimation of absolute mortality radiation risks from typical heart and lung doses in UK breast cancer radiotherapy	22-26

Text S1: Literature searches

a) Terms used in searching Ovid MEDLINE (1 January 2015 to 12 June 2023)

1 exp Breast Neoplasms/rt [Radiotherapy]
2 exp Breast Neoplasms/
3 (breast adj2 (cancer? or neoplas* or tumo?r? or carcinoma? or malignan*)).ti,ab,kf.
4 2 or 3
5 exp Radiotherapy/
6 (radiotherap* or radiation therap*).ti,ab,kf.
7 external beam radiation.ti,ab,kf.
8 irradiat*.ti,ab,kf.
9 brachytherap*.ti,ab,kf.
10 (proton adj2 therap*).ti,ab,kf.
11 5 or 6 or 7 or 9 or 9 or 10
12 4 and 11
13 1 or 12
14 radiation dosage/ or dose-response relationship, radiation/
15 (dose? or dosage? or dosimetr*).ti,ab,kf.
16 14 or 15
17 exp United Kingdom/
18 (national health service* or nhs*).ti,ab,in.
19 (english not ((published or publication* or translat* or written or language* or speak* or literature or citation*) adj5 english)).ti,ab.
20 (gb or "g.b." or britain* or (british* not "british columbia") or uk or "u.k." or united kingdom* or (england* not "new england") or northern ireland* or northern irish* or scotland* or scottish* or ((wales or "south wales") not "new south wales") or welsh*).ti,ab,jw,in.
21 (bangor or "bangor's" or cardiff or "cardiff's" or newport or "newport's " or st asaph or "st asaph's" or st davids or swansea or "swansea's").ti,ab,in.
22 (aberdeen or "aberdeen's" or dundee or "dundee's" or edinburgh or "edinburgh's" or glasgow or "glasgow's" or inverness or (perth not australia*) or ("perth's" not australia*) or stirling or "stirling's").ti,ab,in.
23 (armagh or "armagh's" or belfast or "belfast's" or lisburn or "lisburn's" or londonderry or "londonderry's" or derry or "derry's" or newry or "newry's").ti,ab,in.
24 (bath or "bath's" or ((Birmingham not alabama*) or ("birmingham's" not alabama*) or bradford or "bradford's" or brighton or "brighton's" or bristol or "bristol's" or carlisle* or "carlisle's" or (cambridge not (massachusetts* or boston* or harvard*)) or ("cambridge's" not (massachusetts* or boston* or harvard*)) or (canterbury not zealand*) or ("canterbury's" not zealand*) or chelmsford or "chelmsford's" or chester or "chester's" or chichester or "chichester's" or coventry or "coventry's" or derby or "derby's" or (durham not (carolina* or nc)) or ("durham's" not (carolina* or nc)) or ely or "ely's" or exeter or "exeter's" or gloucester or "gloucester's" or hereford or "hereford's" or hull or "hull's" or lancaster or "lancaster's" or leeds* or leicester or "leicester's" or (lincoln not nebraska*) or ("lincoln's" not nebraska*) or (liverpool not (new south wales* or nsw)) or ("liverpool's" not (new south wales* or nsw)) or ((london not (ontario* or ont or toronto*)) or ("london's" not (ontario* or

ont or toronto*)) or manchester or "manchester's" or (newcastle not (new south wales* or nsw)) or ("newcastle's" not (new south wales* or nsw)) or norwich or "norwich's" or nottingham or "nottingham's" or oxford or "oxford's" or peterborough or "peterborough's" or plymouth or "plymouth's" or portsmouth or "portsmouth's" or preston or "preston's" or ripon or "ripon's" or salford or "salford's" or salisbury or "salisbury's" or sheffield or "sheffield's" or southampton or "southampton's" or st albans or stoke or "stoke's" or sunderland or "sunderland's" or truro or "truro's" or wakefield or "wakefield's" or wells or westminster or "westminster's" or winchester or "winchester's" or wolverhampton or "wolverhampton's" or (worcester not (massachusetts* or boston* or harvard*)) or ("worcester's" not (massachuse tts* or boston* or harvard*)) or (york not ("new york*" or ny or ontario* or ont or toronto*)) or ("york's" not ("new york*" or ny or ontario* or ont or toronto*))))).ti,ab,in. 1735951

25 17 or 18 or 19 or 20 or 21 or 22 or 23 or 24

26 (exp africa/ or exp americas/ or exp antarctic regions/ or exp arctic regions/ or exp asia/ or exp oceania/) not (exp great britain/ or europe/)

27 25 not 26

28 13 and 16 and 27

29 limit 28 to yr="2015 -Current"

30 exp animals/ not humans/

31 29 not 30

b) Terms used in searching Embase (1 January 2015 to 12 June 2023)

1 *breast cancer/rt or *breast radiotherapy/
2 exp *breast cancer/
3 (breast adj2 (cancer? or neoplas* or tumo?r? or carcinoma? or malignan*)).ti,ab,kf.
4 2 or 3
5 *cancer radiotherapy/ or *adjuvant radiotherapy/ or *irradiation/
6 (radiotherap* or radiation therap*).ti,ab,kf.
7 external beam radiation.ti,ab,kf.
8 irradiat*.ti,ab,kf.
9 brachytherap*.ti,ab,kf.
10 (proton adj2 therap*).ti,ab,kf.
11 5 or 6 or 7 or 8 or 9 or 10
12 4 and 11
13 1 or 12
14 radiation depth dose/ or radiation dose escalation/ or exp radiation dose fractionation/ or radiation dose reduction/ or exp radiotherapy dosage/
15 (dose or dosage? or dosing or dosimetr*).ti,ab,kf.
16 14 or 15
17 13 and 16
18 exp United Kingdom/
19 (national health service* or nhs*).ti,ab,in.
20 (english not ((published or publication* or translat* or written or language* or speak* or literature or citation*) adj5 english)).ti,ab.
21 (gb or "g.b." or britain* or (british* not "british columbia") or uk or "u.k." or united kingdom* or (england* not "new england") or northern ireland* or northern irish* or scotland* or scottish* or ((wales or "south wales") not "new south wales") or welsh*).ti,ab,jw,in.
22 (bangor or "bangor's" or cardiff or "cardiff's" or newport or "newport's " or st asaph or "st asaph's" or st davids or swansea or "swansea's").ti,ab,in.
23 (aberdeen or "aberdeen's" or dundee or "dundee's" or edinburgh or "edinburgh's" or glasgow or "glasgow's" or inverness or (perth not australia*) or ("perth's" not australia*) or stirling or "stirling's").ti,ab,in.
24 (armagh or "armagh's" or belfast or "belfast's" or lisburn or "lisburn's" or londonderry or "londonderry's" or derry or "derry's" or newry or "newry's").ti,ab,in.
25 (bath or "bath's" or ((Birmingham not alabama*) or ("birmingham's" not alabama*) or bradford or "bradford's" or brighton or "brighton's" or bristol or "bristol's" or carlisle* or "carlisle's" or (cambridge not (massachusetts* or boston* or harvard*)) or ("cambridge's" not (massachusetts* or boston* or harvard*)) or (canterbury not zealand*) or ("canterbury's" not zealand*) or chelmsford or "chelmsford's" or chester or "chester's" or chichester or "chichester's" or coventry or "coventry's" or derby or "derby's" or (durham not (carolina* or nc)) or ("durham's" not (carolina* or nc)) or ely or "ely's" or exeter or "exeter's" or gloucester or "gloucester's" or hereford or "hereford's" or hull or "hull's" or lancaster or "lancaster's" or leeds* or leicester or "leicester's" or (lincoln not nebraska*) or ("lincoln's" not nebraska*) or (liverpool not (new south wales* or nsw)) or ("liverpool's" not (new south wales* or nsw)) or ((london not (ontario* or ont or toronto*)) or ("london's" not (ontario* or

ont or toronto*)) or manchester or "manchester's" or (newcastle not (new south wales* or nsw)) or ("newcastle's" not (new south wales* or nsw)) or norwich or "norwich's" or nottingham or "nottingham's" or oxford or "oxford's" or peterborough or "peterborough's" or plymouth or "plymouth's" or portsmouth or "portsmouth's" or preston or "preston's" or ripon or "ripon's" or salford or "salford's" or salisbury or "salisbury's" or sheffield or "sheffield's" or southampton or "southampton's" or st albans or stoke or "stoke's" or sunderland or "sunderland's" or truro or "truro's" or wakefield or "wakefield's" or wells or westminster or "westminster's" or winchester or "winchester's" or wolverhampton or "wolverhampton's" or (worcester not (massachusetts* or boston* or harvard*)) or ("worcester's" not (massachuse tts* or boston* or harvard*)) or (york not ("new york*" or ny or ontario* or ont or toronto*)) or ("york's" not ("new york*" or ny or ontario* or ont or toronto*))))).ti,ab,in.

26 18 or 19 or 20 or 21 or 22 or 23 or 24 or 25

27 (exp africa/ or exp asia/ or exp Pacific Islands/ or "Australia and New Zealand"/ or "arctic and antarctic"/ or exp north america/ or exp "south and central america"/) not (exp United Kingdom/ or Europe/)

28 26 not 27

29 17 and 28

30 (rat or rats or mouse or mice or swine or porcine or murine or sheep or lambs or pigs or piglets or rabbit or rabbits or cat or cats or dog or dogs or cattle or bovine or monkey or monkeys or trout or marmoset$1).ti. and animal experiment/

31 Animal experiment/ not (human experiment/ or human/)

32 30 or 31

33 29 not 32

34 limit 33 to yr="2015 -Current"

35 conference*.pt. or conference abstract/

36 34 not 35

Text S2. Definitions used for data extraction

**Study design**	**Dosimetry**	A study in which radiotherapy plans were simulated for research purposes and planned doses were not delivered to patients.
	**Dosimetry & Observational**	A study including some radiotherapy plans delivered to patients in routine practice and some plans simulated for research.
	**Observational/ audit**	A study retrospectively reporting doses delivered to patients in routine clinical practice.
	**Interventional**	A prospective clinical trial reporting doses delivered to trial patients randomised between different radiotherapy practices.
**Treatment planning methods**	**3D CRT**	3D conformal radiotherapy using multi-CT patient contour slices with shaped fields
	**3D CRT FIF**	Multiple segments used (field-in-field)
	**IMRT**	Intensity modulated radiotherapy using multi-CT patient contour slices with inverse-planned IMRT (assumes shaped fields and multiple segments as standard)
	**Hybrid**	A combination of 3D CRT +/- FIF tangents and inverse planned rotational IMRT
	**PBT**	Proton beam therapy
	**Auto.**	Plans automatically created by running a computer programme.
	**MF**	Manual flash used within plan to incorporate ‘skin flash’ in the radiotherapy field apertures. This is usually done by including around 2cm of air surrounding the breast within the field to ensure irradiation of all breast tissue should the target increase in size from the time of the planning CT.
	**RO-OM**	Robust optimisation for organ motion used in creating the radiotherapy plan. This is done by optimising over a range of simulated CTs representing worst-case scenario plan changes. For example, changes in target breast tissue size from the time of planning CT may be simulated by Deformable image Registration based on user-defined organ motion.
**Radiotherapy technique**	**Tangents**	Fixed gantry angles, tangential breast/chest wall fields +/- matched direct SF/axilla direct field. This may include tangents, wide tangents, partially wide tangents.
	**Tangents FIF**	Fixed gantry angles, tangential breast/chest wall fields +/- matched direct SF/axilla direct field. Multiple segments used (FIF)
	**IMRT static**	Multiple static direct or oblique fields of IMRT (step and shoot)
	**IMRT rotational**	Dynamic fields using a series of arcs, gantry rotates during treatment, using Tomotherapy (helical delivery) or a linear accelerator (e.g. volumetric modulated arc therapy)
	**IMRT PBS**	IMRT Pencil beam scanning proton beam therapy

Abbreviations: Auto.=automated, 3D-CRT=3-dimensional conformal radiotherapy, FIF=Field-in-Field, IMRT=Intensity-modulated radiation therapy, PBT=Proton beam therapy, PBS=Pencil beam scanning, MF=Manual flash, SF=supraclavicular fossa lymph nodes, RO-OM=Robust optimisation-organ motion.

Figure S1: Process of study identification

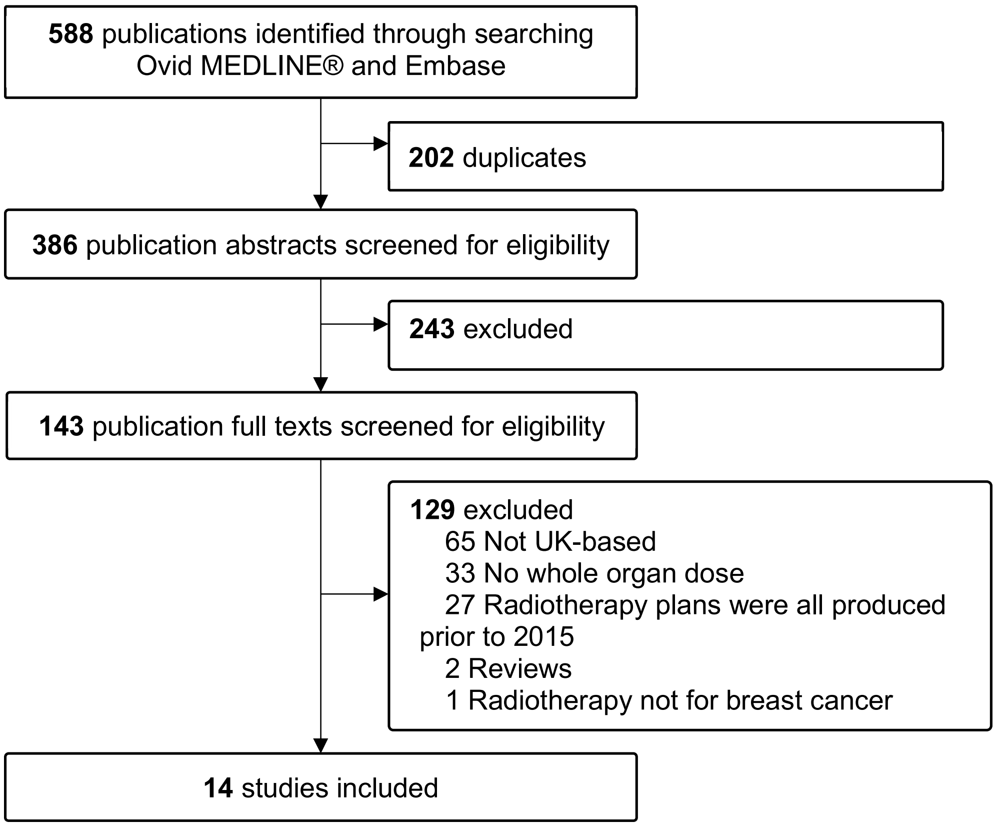

Text S3: References for included studies

Coles CE, Haviland JS, Kirby AM, et al. Dose-escalated simultaneous integrated boost radiotherapy in early breast cancer (IMPORT HIGH): a multicentre, phase 3, non-inferiority, open-label, randomised controlled trial. *Lancet* 2023; **401**(10394): 2124-37. https://dx.doi.org/10.1016/S0140-6736(23)00619-0

Dunlop A, Colgan R, Kirby A, Ranger A, Blasiak-Wal I. Evaluation of organ motion-based robust optimisation for VMAT planning for breast and internal mammary chain radiotherapy. *Clinical and Translational Radiation Oncology* 2019; **16**: 60-6. https://dx.doi.org/10.1016/j.ctro.2019.04.004

Gleeson I. Comparing the robustness of different skin flash approaches using wide tangents, manual flash VMAT, and simulated organ motion robust optimization VMAT in breast and nodal radiotherapy. *Medical dosimetry : official journal of the American Association of Medical Dosimetrists* 2022; **47**(3): 264-72. https://dx.doi.org/10.1016/j.meddos.2022.04.004

Gleeson I, Bolger N, Chun H, et al. Implementation of automated personalised breast radiotherapy planning techniques with scripting in Raystation. *The British journal of radiology* 2023; **96**(1144): 20220707. https://dx.doi.org/10.1259/bjr.20220707

Ledsom D, Reilly AJ, Probst H. Assessment of deep inspiration breath hold (DIBH) amplitude and reduction in cardiac dose in left breast cancer patients. *Radiography* 2018; **24**(2): 98-103. https://dx.doi.org/10.1016/j.radi.2017.11.005

Locke I, Drinkwater K. Implementation of Royal College of Radiologists Consensus Statements and National Institute for Health and Care Excellence Guidance: Breast Radiotherapy Practice in the UK. *Clinical Oncology* 2021; **33**(7): 419-26. https://dx.doi.org/10.1016/j.clon.2021.01.012

Mitchell RA, Wai P, Colgan R, Kirby AM, Donovan EM. Improving the efficiency of breast radiotherapy treatment planning using a semi-automated approach. *Journal of applied clinical medical physics* 2017; **18**(1): 18-24. https://dx.doi.org/10.1002/acm2.12006

Murphy SBD, Drury-Smith H. Which simultaneous-integrated boost (SIB) intensity-modulated radiotherapy (IMRT) technique is dosimetrically superior in the treatment of breast cancer; volumetric-modulated arc therapy (VMAT) or fixed field (ff) IMRT? *Journal of Radiotherapy in Practice* 2017; **16**(3): 272-9. https://dx.doi.org/10.1017/S1460396917000164

Ranger A, Dunlop A, Grimwood A, et al. Voluntary versus ABC breath-hold in the context of VMAT for breast and locoregional lymph node radiotherapy including the internal mammary chain. *Clinical and Translational Radiation Oncology* 2021; **27**: 164-8. https://dx.doi.org/10.1016/j.ctro.2021.02.003

Ranger A, Dunlop A, Hansen VN, et al. A Randomised Phase II Clinical Trial Comparing the Deliverability and Acute Toxicity of Wide Tangent versus Volumetric Modulated Arc Therapy to the Breast and Internal Mammary Chain. *Clinical oncology* 2022; **34**(8): 526-33. https://dx.doi.org/10.1016/j.clon.2022.03.020

Ranger A, Dunlop A, Hutchinson K, et al. A Dosimetric Comparison of Breast Radiotherapy Techniques to Treat Locoregional Lymph Nodes Including the Internal Mammary Chain. *Clinical Oncology* 2018; **30**(6): 346-53.
https://dx.doi.org/10.1016/j.clon.2018.01.017

Ranger A, Dunlop A, Shah P, et al. Evaluation of a Novel Field-placement Algorithm for Locoregional Breast Cancer Radiotherapy Including the Internal Mammary Chain. *Clinical oncology* 2019; **31**(1): 25-33.
https://dx.doi.org/10.1016/j.clon.2018.06.014

Welgemoed C, Coughlan S, McNaught P, Gujral D, Riddle P. A dosimetric study to improve the quality of nodal radiotherapy in breast cancer. *BJR Open* 2021; **2**(1): 20210013.
https://dx.doi.org/10.1259/bjro.20210013

Wickers S, Thould C, Keeble J, et al. Identifying Surrogates for Heart and Ipsilateral Lung Dose to Guide Field Placement and Treatment Modality Selection during Virtual Simulation of Breast Radiotherapy. *Clinical oncology* 2021; **33**(4): 224-9.
https://dx.doi.org/10.1016/j.clon.2020.12.00

Table S1. Studies reporting whole heart doses from radiotherapy for early breast cancer published 2015-2023

Author & Year of publication Study location*	Study design†	Patients	CT scans	Individual dose estimates‡	Individual dose estimates delivered to patients (%)
Gleeson 2023 Cambridge	Dosimetry & Observational	41	41	82	41 (50)
Coles 2023 Multi-centre	Interventional	1958	1958	1958	1958 (100)
Ranger 2022 London	Interventional	19	19	19	19 (100)
Gleeson 2022 Cambridge	Dosimetry & Observational	10	10	40	10 (25)
Wickers 2021 London	Dosimetry & Observational	59	59	59	50 (85)
Locke 2021 Multi-centre	Observational/ Audit	355	355	355	355 (100)
Ranger 2021 London	Interventional	16	32	32	32 (100)
Dunlop 2019 London	Dosimetry	5	5	10	0 (0)
Ranger 2019 Unspecified	Dosimetry	20	20	40	0 (0)
Ranger 2018 Multi-centre	Dosimetry	14	14	98	0 (0)
Ledsom 2018 Liverpool	Dosimetry	30	60	60	0 (0)
Murphy 2017 Unspecified	Dosimetry	30	30	60	0 (0)
Mitchell 2017 London	Dosimetry & Observational	40	40	80	40 (50)
**ALL STUDIES**		**2597**	**2643**	**2893**	**2505 (87)**

Footnotes

*Years during which each study was conducted were available for four of the thirteen studies: Coles 2023 was conducted 2009-2015, Ranger 2022 was conducted 2017-2018, Wickers 2021 was completed in 2020 and Locke 2021 was completed in 2019.

† See Text S2 for definitions.

‡This refers to the number of individual dose estimates contributing to each reported average dose. In some studies patients had more than one CT planning scan, and more than one radiotherapy plan may have been applied to each CT planning scan. Doses to all organs at risk were not always estimated from all plans.

There was no published information within included studies on the number of patients with unusual anatomy.

Abbreviations: CT=computed tomography

Table S2. Mean whole heart doses (Gy) and radiotherapy descriptions from thirteen UK breast cancer radiotherapy studies published during 2015-2023

		Description of radiotherapy										Whole heart dose (Gy)[#]		
Author & year of publication*	Location	Treatment planning[†]	Radiotherapy technique[†]	Beam energy (MV)[‡]	Breath control	Laterality	Clinical targets irradiated[§]	Target dose (Gy)	Fraction	Boost	No. dose estimates[¶]	Mean/ median	SD	Range
Coles 2023	Multi-centre	IMRT/3D CRT	IMRT static	4-15	DIBH/FB	L	Br + Ax/SF	56	23	Seq.	316	2.4	NS	NS
		IMRT/3D CRT	IMRT static	4-15	DIBH/FB	R	Br + Ax/SF	56	23	Seq.	316	1.0	NS	NS
		IMRT	IMRT static	4-15	DIBH/FB	L	Br + Ax/SF	48	15	Sim.	319	2.7	NS	NS
		IMRT	IMRT static	4-15	DIBH/FB	R	Br + Ax/SF	48	15	Sim.	340	1.3	NS	NS
		IMRT	IMRT static	4-15	DIBH/FB	L	Br + Ax/SF	53	15	Sim.	340	2.9	NS	NS
		IMRT	IMRT static	4-15	DIBH/FB	R	Br + Ax/SF	53	15	Sim.	327	1.4	NS	NS
Dunlop 2019	London	IMRT	IMRT rotational	6	DIBH, V	L	Br ± Ax/SF + IM	40	15	NS	5	3.9	NS	NS
		IMRT, RO	IMRT rotational	6	DIBH, V	L	Br ± Ax/SF + IM	40	15	NS	5	3.8	NS	NS
Gleeson 2022	Cambridge	IMRT	IMRT static	6-10	DIBH	L/R	Br/CW ± Ax/SF + IM	40	15	NS	10	1.2	0.3	NS
		IMRT	IMRT rotational	6	DIBH	L/R	Br/CW ± Ax/SF + IM	40	15	NS	10	2.0	0.7	NS
		IMRT, MF	IMRT rotational	6	DIBH	L/R	Br/CW ± Ax/SF + IM	40	15	NS	10	2.0	0.7	NS
		IMRT, RO	IMRT rotational	6	DIBH	L/R	Br/CW ± Ax/SF + IM	40	15	NS	10	2.0	0.7	NS
Gleeson 2023	Cambridge	Hybrid	IMRT rotational	6	DIBH/FB	L/R	Br only	48	15	Sim.	21	0.9	0.6	0.2-2.8
		Hybrid, automated	IMRT rotational	6	DIBH/FB	L/R	Br only	48	15	Sim.	21	0.9	0.6	0.2-2.5
		IMRT	IMRT rotational	6	DIBH/FB	L/R	Br ± Ax/SF + IM	40	15	No boost	20	2.8	0.6	1.6-3.9
		IMRT, automated	IMRT rotational	6	DIBH/FB	L/R	Br ± Ax/SF + IM	40	15	No boost	20	3.0	0.4	2.2-3.7
Ledsom 2018	Liverpool	3D CRT FIF	Tangents FIF	6-10	FB	L	Br + SF	40	15	NS	30	3.4	NS	1.8-5.6
		3D CRT FIF	Tangents FIF	6-10	DIBH, V	L	Br + SF	40	15	NS	30	1.3	NS	0.6-2.2
Locke 2021	Multi-centre	NS	NS	NS	DIBH/FB	L	NS	NS	NS	NS	355	1.2	NS	-8.72
Mitchell 2017	London	IMRT	IMRT static	6-10	NS	L/R	Br + SF	40	15	NS	40	0.8	NS	0.3-2.4
		IMRT, automated	IMRT static	6-10	NS	L/R	Br + SF	40	15	NS	40	0.8	NS	0.3-2.5
Murphy 2017	NS	Hybrid	IMRT rotational	NS	DIBH, V/FB	L	Br only	48	15	Sim.	15	1.4	0.4	NS
		Hybrid	IMRT rotational	NS	DIBH, V/FB	R	Br only	48	15	Sim.	15	0.9	0.2	NS
		IMRT	IMRT static	NS	DIBH, V/FB	L	Br only	48	15	Sim.	15	1.5	0.5	NS
		IMRT	IMRT static	NS	DIBH, V/FB	R	Br only	48	15	Sim.	15	1.0	0.3	NS

Table S2 continued on the next page.

Table S2 continued from the previous page.

Author & year of publication*	Location	Treatment planning†	Radiotherapy technique†	Beam energy (MV)‡	Breath control	Laterality	Clinical targets irradiated§	Target dose (Gy)	Fraction	Boost	No. dose estimates¶	Mean/ median	SD	Range
		Description of radiotherapy										Whole heart dose (Gy)#		
Ranger 2018	Multi-centre	3D CRT FIF	Tangents FIF	6-10	DIBH, V	L	Br/CW ± Ax/SF + IM	40	15	NS	14	2.5	1.2	NS
		3D CRT FIF	Tangents FIF	6-10	FB	L	Br/CW ± Ax/SF + IM	40	15	NS	14	5.3	1.0	NS
		IMRT	IMRT rotational	NS	DIBH, V	L	Br/CW ± Ax/SF + IM	40	15	NS	14	2.6	1.0	NS
		IMRT	IMRT rotational	NS	FB	L	Br/CW ± Ax/SF + IM	40	15	NS	14	4.5	1.3	NS
		IMRT	IMRT rotational	NS	FB	L	Br/CW ± Ax/SF + IM	40	15	NS	14	6.4	1.4	NS
		PBT	IMRT pencil beam scanning	Proton NS	DIBH, V	L	Br/CW ± Ax/SF + IM	40	15	NS	14	0.5	0.1	NS
		PBT	IMRT pencil beam scanning	Proton NS	FB	L	Br/CW ± Ax/SF + IM	40	15	NS	14	1.0	0.1	NS
Ranger 2019	NS	3D CRT	Tangents	6-10	DIBH, V	L	Br/CW ± Ax/SF + IM	40	NS	NS	20	1.5	NS	1.3-4.6
		3D CRT	Tangents	6-10	DIBH, V	L	Br/CW ± Ax/SF + IM	40	NS	NS	20	1.8	NS	1.4-4.2
Ranger 2021	London	IMRT	IMRT rotational	NS	DIBH, V	L/R	Br/CW ± Ax/SF + IM	NS	15	NS	16	4.2	1.3	NS
		IMRT	IMRT rotational	NS	DIBH, ABC	L/R	Br/CW ± Ax/SF + IM	NS	15	NS	16	4.4	1.3	NS
Ranger 2022	London	3D CRT FIF	Tangents FIF	6-10	DIBH,V	L	Br/CW ± Ax/SF + IM	40	15	NS	9	2.6	1.2	NS
		IMRT	IMRT rotational	NS	DIBH, V/FB	L	Br/CW ± Ax/SF + IM	40	15	NS	10	3.6	0.7	NS
Wickers 2021	London	3D CRT FIF	Tangents FIF	6-10	FB	L	Br/CW only	40	15	NS	50	2.3	NS	1.2-8.0
		3D CRT FIF	Tangents FIF	6-10	DIBH	L	Br/CW + SF	40	15	NS	5	2.6	NS	1.4-4.0
		3D CRT FIF	Tangents FIF	6-10	DIBH	L	Br/CW ± Ax/SF + IM	40	15	NS	4	2.0	NS	1.5-4.7

Footnotes

Thirteen of the fourteen studies included in the review reported whole heart doses.

Average mean whole heart doses across all studies and subgroups of studies were weighted by the number of individual dose estimates contributing to the study mean doses.

*See Text S3 for references.

†See Text S2 for definitions.

Where specified, all patients were planned in the supine position. Six studies did not specify patient position (Murphy 2017, Mitchell 2017, Ledsom 2018, Ranger 2019, Dunlop 2019, Locke 2021).

‡PBT uses proton beams, all other planning types specified the use of photons. Only one study did not specify radiotherapy beam type (Locke 2021).

§When analysing dose by clinical targets irradiated, it was assumed that if a target was not specified, it was not irradiated. None of the included studies reported radiotherapy to the following targets: partial breast, breast reconstructions with prosthesis. One study (Ranger 2018) specified inclusion of three patients with autologous breast reconstruction.

¶This refers to the number of individual dose estimates contributing to each published mean dose. It was reported in all studies, with the exception of Coles 2023. For this study, the numbers of individual patients for whom dosimetry information was reported within each trial group were 632, 659 and 667. Of these patients, the numbers who received radiotherapy for left and right breast cancer were unavailable and therefore estimated by applying the percentage of patients with right and left breast cancers in each of the three trial groups to the numbers of patients with available dosimetry information.

#Details of heart contouring guidelines used to delineate organs at risk were published by Coles 2023, Gleeson 2023 and Ledsom 2018. All three studies stated the heart contour inferiorly extends to above the diaphragm, and superiorly extends to below the pulmonary arch.

Abbreviations: ABC=active breathing control, Ax=axilla lymph nodes, Br=breast, CW=chest wall, DIBH=deep inspiratory breath hold, FB=free breathing, Gy=gray, IM=internal mammary lymph nodes, L=left radiotherapy, MF=manual flash, MV=megavoltage, R=right radiotherapy, RO=robust optimisation, SD=standard deviation, Seq.=sequential, SF=supraclavicular fossa lymph nodes, Sim.=simultaneous, V=voluntary, NS=not specified.

Table S3. Studies reporting ipsilateral and contralateral lung doses from radiotherapy for early breast cancer published during 2015-2023

Author & Year of publication Study location*	Study design†	Patients	CT scans	Individual dose estimates‡	Individual dose estimates delivered to patients (%)
Gleeson 2023 Cambridge	Dosimetry & Observational	41	41	82	41 (50)
Coles 2023 Multi-centre	Interventional	1958	1958	1958	1958 (100)
Gleeson 2022 Cambridge	Dosimetry & Observational	10	10	40	10 (25)
Dunlop 2019 London	Dosimetry	5	5	10	0 (0)
Murphy 2017 Unspecified	Dosimetry	30	30	60	0 (0)
Mitchell 2017 London	Dosimetry & Observational	40	40	80	40 (50)
**ALL STUDIES**		**2084**	**2084**	**2230**	**2049 (92)**

Footnotes

*Years during which the study was conducted was available for one of the six studies: Coles 2023 was conducted 2009-2015.

† See Text S2 for definitions.

‡This refers to the number of individual dose estimates contributing to each reported average dose. In some studies patients had more than one CT planning scan, and more than one radiotherapy plan may have been applied to each CT planning scan. Doses to all organs at risk were not always estimated from all plans.

There was no published information within included studies on the number of patients with unusual anatomy.

Abbreviations: CT=computed tomography

Table S4. Estimated mean whole lung doses (Gy) and radiotherapy descriptions from six UK breast cancer radiotherapy studies published during 2015-2023

		Description of radiotherapy										Lung dose (Gy) Ipsilateral			Lung dose (Gy) Contralateral			Lung dose (Gy) Whole¶	
Author & year of publication*	Location	Treatment planning†	Radiotherapy technique†	Beam energy (MV)	Breath control	Laterality	Clinical targets irradiated‡	Target dose (Gy)	Fraction	Boost	No. dose estimates§	Mean/ median	SD	Range	Mean/ median	SD	Range	Mean/ median	Range
Coles 2023	Multi-centre	IMRT/ 3D CRT	IMRT static	4-15	DIBH/FB	L/R	Br + Ax/SF	56	23	Seq.	632	5.2	NS	NS	0.4	NS	NS	2.8	NS
		IMRT	IMRT static	4-15	DIBH/FB	L/R	Br + Ax/SF	48	15	Sim.	659	5.4	NS	NS	0.5	NS	NS	3.0	NS
		IMRT	IMRT static	4-15	DIBH/FB	L/R	Br + Ax/SF	53	15	Sim.	667	5.7	NS	NS	0.6	NS	NS	3.2	NS
Dunlop 2019	London	IMRT	IMRT rotational	6	DIBH, V	L	Br ± Ax/SF + IM	40	15	NS	5	13.6	NS	NS	3.3	NS	NS	8.5	NS
		IMRT, RO	IMRT rotational	6	DIBH, V	L	Br ± Ax/SF + IM	40	15	NS	5	13.6	NS	NS	3.3	NS	NS	8.5	NS
Gleeson 2022	Cambridge	IMRT	IMRT static	6-10	DIBH	L/R	Br/CW ± Ax/SF + IM	40	15	NS	10	10.0	1.7	NS	0.6	0.0	NS	5.3	NS
		IMRT	IMRT rotational	6	DIBH	L/R	Br/CW ± Ax/SF + IM	40	15	NS	10	12.1	0.6	NS	1.0	0.2	NS	6.6	NS
		IMRT, MF	IMRT rotational	6	DIBH	L/R	Br/CW ± Ax/SF + IM	40	15	NS	10	12.2	0.6	NS	1.0	0.2	NS	6.6	NS
		IMRT, RO	IMRT rotational	6	DIBH	L/R	Br/CW ± Ax/SF + IM	40	15	NS	10	12.6	0.3	NS	1.3	1.0	NS	7.0	NS
Gleeson 2023	Cambridge	Hybrid	IMRT rotational	6	DIBH/FB	L/R	Br only	48	15	Sim.	21	3.7	0.6	2.8-5.4	0.3	0.1	0.1-0.6	2.0	1.5-3.0
		Hybrid, automated	IMRT rotational	6	DIBH/FB	L/R	Br only	48	15	Sim.	21	3.7	0.6	2.7-5.2	0.3	0.1	0.1-0.6	2.0	1.4-2.9
		IMRT	IMRT rotational	6	DIBH/FB	L/R	Br ± Ax/SF + IM	40	15	No boost	20	13.5	1.1	12.4-16.4	1.2	0.3	0.9-2.2	7.4	6.7-9.3
		IMRT, automated	IMRT rotational	6	DIBH/FB	L/R	Br ± Ax/SF + IM	40	15	No boost	20	13.9	1.6	12.3-18.3	1.2	0.1	1.1-1.6	7.6	6.7-10.0
Mitchell 2017	London	IMRT	IMRT static	6-10	NS	L/R	Br ±SF	40	15	NS	40	4.5	NS	1.7-9.7	0.2	NS	0.1-0.4	2.4	0.9-5.1
		IMRT, automated	IMRT static	6-10	NS	L/R	Br ±SF	40	15	NS	40	4.6	NS	1.7-9.7	0.2	NS	0.1-0.4	2.4	0.9-5.1
Murphy 2017	NS	Hybrid	IMRT rotational	NS	DIBH, V/FB	L/R	Br only	48	15	Sim.	30	5.5	0.9	NS	0.5	0.1	NS	3.0	NS
		IMRT	IMRT static	NS	DIBH, V/FB	L/R	Br only	48	15	Sim.	30	5.4	0.8	NS	0.5	0.1	NS	3.0	NS

Footnotes

Six of the fourteen studies included in the review reported ipsilateral and contralateral lung doses.
Average mean whole lung doses across all studies and subgroups of studies were weighted by the number of individual dose estimates contributing to study mean doses.
All mean whole lung dose estimates included some radiotherapy plans for left and some for right cancers, apart from Dunlop 2019 which reported on radiotherapy for left breast cancer only.
*See Text S3 for references.
†See Text S2 for definitions.
Where specified, all patients were planned in the supine position. Three studies did not specify patient position (Murphy 2017, Mitchell 2017, Dunlop 2019).
‡When analysing dose by clinical targets irradiated, it was assumed that if a target was not specified, it was not irradiated. None of the included studies reported radiotherapy to the following targets: partial breast, breast reconstructions.
§This refers to the number of individual dose estimates contributing to each published mean dose.
¶Whole lung dose was estimated by taking the average of the reported mean ipsilateral and contralateral lung doses. There were no published details of contouring guidelines used to delineate the lungs. One study described using auto-contouring to delineate the lungs (Mitchell 2017).
Abbreviations: Ax=axilla lymph nodes, Br=breast, CW=chest wall, DIBH=deep inspiratory breath hold, FB=free breathing, Gy=gray, IM=internal mammary lymph nodes, L=left radiotherapy, MF=manual flash, MV=megavoltage, R=right radiotherapy, RO=robust optimisation, SD=standard deviation, Seq.=sequential, SF=supraclavicular fossa lymph nodes, Sim.=simultaneous, V=voluntary, NS=not specified.

Table S5. Studies reporting whole oesophagus doses from radiotherapy for early breast cancer published during 2015-2023

Author & Year of publication Study location*	Study design†	Patients	CT scans	Individual dose estimates‡	Individual dose estimates delivered to patients (%)
Gleeson 2023 Cambridge	Dosimetry & Observational	20	20	40	20 (50)
Welgemoed 2021 London	Dosimetry	12	12	36	0 (0)
**ALL STUDIES**		**32**	**32**	**76**	**20 (26)**

Footnotes

*Years during which studies were conducted were unspecified for both studies.

† See Text S2 for definitions.

‡This refers to the number of individual dose estimates contributing to each reported average dose. In some studies patients had more than one CT planning scan, and more than one radiotherapy plan may have been applied to each CT planning scan. Doses to all organs at risk were not always estimated from all plans. There was no published information within included studies on the number of patients with unusual anatomy.

Abbreviations: CT=computed tomography

Table S6. Mean whole oesophagus doses (Gy) and radiotherapy descriptions from two UK breast cancer radiotherapy studies published during 2015-2023

Author & year of publication*	Location	Description of RT regimen									No. doses estimates§	Whole oesophagus dose (Gy)¶		
		Treatment planning†	Radiotherapy technique†	Beam energy (MV)	Breath control	Laterality	Clinical targets irradiated‡	Target dose (Gy)	Fraction	Boost		Mean	SD	Range
Gleeson 2023	Cambridge	IMRT	IMRT rotational	6	DIBH/FB	L/R	Br ± Ax/SF + IM	40	15	No	20	5.4	2.0	1.9-10.0
		IMRT, automated	IMRT rotational	6	DIBH/FB	L/R	Br ± Ax/SF + IM	40	15	No	20	6.1	1.8	2.4-9.7
Welgemoed 2021	London	3D CRT FIF	Tangents FIF	6-10	NS	NS	Br/CW + Ax/SF	40	15	NS	12	1.2	0.3	NS
		3D CRT FIF	Tangents FIF	6-10	NS	NS	Br/CW + Ax/SF	40	15	NS	12	2.0	2.0	NS
		3D CRT FIF	Tangents FIF	6-10	NS	NS	Br/CW + Ax/SF	40	15	NS	12	1.0	0.4	NS

Footnotes

Two of fourteen studies included in the review reported doses to the whole oesophagus.
Average mean whole oesophagus doses across all studies and subgroups of studies were weighted by the number of individual dose estimates contributing to study mean doses.
Mean whole oesophagus doses included some radiotherapy plans for left and some for right cancers in one study (Gleeson 2023). The other study did not specify laterality of radiotherapy (Welgemoed 2021).
*See Text S3 for references.
†See Text S2 for definitions.
All patients were planned in the supine position.
‡When analysing dose by clinical targets irradiated, it was assumed that if a target was not specified, it was not irradiated. None of the included studies reported radiotherapy to the following targets: partial breast, breast reconstructions.
§This refers to the number of individual dose estimates contributing to each published mean dose.
¶There were no published details of contouring guidelines used to delineate the oesophagus.
Abbreviations: Ax=axilla lymph nodes, Br=breast, CW=chest wall, DIBH=deep inspiratory breath hold, FB=free breathing, Gy=gray, IM=internal mammary lymph nodes, L=left radiotherapy, MV=megavoltage, R=right radiotherapy, SD=standard deviation, SF=supraclavicular fossa lymph nodes, V=voluntary, NS=not specified.

Text S4: Estimated radiation doses to heart, lungs and oesophagus from breast cancer radiotherapy in the Oxford Cancer Centre, 2018

Introduction

Estimated typical organ doses from modern UK breast cancer radiotherapy using published data may not represent routine clinical practice. Published dosimetry and interventional studies may report lower doses to organs than those delivered in routine clinical practice because the radiotherapy in such studies may use more advanced radiotherapy techniques than those available routinely. Even centres publishing routinely-collected observational/audit data may report lower doses than typical because these centres may be more engaged with practices to minimise organ doses. The extent to which our average published organ doses represent routine clinical practice is uncertain.

Here we present whole organ doses to the heart, lungs and oesophagus from 50 patients irradiated for early breast cancer at the Oxford Cancer Centre during 2018, outside of the setting of any clinical study. We then compare these with summary doses from published studies during 2015-2023.

Methods

Data analysis methods used to estimate typical doses from modern UK breast cancer radiotherapy using published doses were repeated using individual patient data obtained from an audit conducted at the Oxford Cancer Centre during 2018. The purpose of the audit was to assess compliance with national standards for reducing risks of breast cancer radiotherapy.[1-2] The local research ethics committee at Oxford University Hospitals NHS Foundation Trust approved the audit and it was registered with the local Clinical Audit Lead.

Patient selection

Radiotherapy CT scans and plans were selected from 50 consecutive women on the CT planning database with radiotherapy for early breast cancer at the Oxford Cancer Centre during May and June 2018. They all consented for their data to be used for research.

Contouring organs on CT planning scans

For each patient's radiotherapy CT scan, the heart, lungs and oesophagus were retrospectively reviewed and contoured using published contouring guidelines.[3-4]

Data extraction

From each patient CT radiotherapy plan the following information was extracted: breast cancer laterality, radiotherapy clinical targets irradiated and whole (ie mean) doses (Gy) to heart, both lungs combined and oesophagus.

Data analysis

The dose data were anonymised and summarised. Whole organ doses (Gy) were categorised first by breast cancer laterality ("left" or "right"), and then by clinical targets irradiated ("breast or chest wall only", "breast or chest wall +axilla/supraclavicular fossa lymph nodes", "breast or chest wall ±axilla/supraclavicular fossa + internal mammary lymph nodes"). Typical doses to organs were estimated by calculating the mean whole organ dose for each category of laterality and clinical targets irradiated.

These estimates of mean whole organ doses from 50 patients irradiated for early breast cancer at Oxford Cancer Centre during 2018 were then compared with the average mean whole organ doses from published studies of UK radiotherapy, 2015-2023.

Results

Of the 50 women irradiated for early breast cancer at Oxford Cancer Centre during 2018, 30 (60%) had left and 20 (40%) had right breast cancer. Radiotherapy to the breast or chest wall was received by 35 of the 50 (70%) women. Breast or chest wall + axilla/supraclavicular fossa lymph nodes radiotherapy was received by 14 (28%) of the women (Table S7). Radiotherapy targeting the internal mammary nodes was received by one (2%) patient with right breast cancer. No patient with left breast cancer received radiotherapy to the internal mammary lymph nodes.

The mean whole heart dose for all 50 women was 1.0 Gy (range 0.2-2.9) (Table S7a). Mean whole heart dose was higher for women with left compared with right breast cancer (left 1.3 Gy vs right 0.4 Gy). It was slightly higher for women who received radiotherapy that included the supraclavicular fossa or axillary nodes compared with women receiving radiotherapy to the breast or chest wall only (left: nodes 1.5 Gy vs no nodes 1.2 Gy, right: nodes 0.5 Gy vs no nodes 0.4 Gy). For all combinations of laterality and targets, mean whole heart doses were lower in the audit compared with average mean whole heart doses in the review of published doses.

The mean whole lung dose for all 50 women in the audit was 3.8 Gy (range 2.0-6.8) (Table S7b). For women receiving radiotherapy that included the supraclavicular fossa or axillary nodes, the mean whole lung dose was higher than for women receiving breast or chest wall radiotherapy only (nodes 4.6 Gy vs no nodes 3.5 Gy). Target-specific average mean whole lung doses in the review of published data were compared with mean whole lung doses in the audit: for breast/chest wall only and for breast or chest wall +axilla/supraclavicular fossa lymph nodes, lung dose was higher in the audit than in the review of published doses (breast/chest wall: audit 3.5 Gy, review 2.6 Gy; breast or chest wall +axilla/supraclavicular fossa lymph nodes: audit 4.6 Gy, review 3.0 Gy). Only one patient in the audit received radiotherapy to the internal mammary chain.

For whole oesophagus, the mean dose for all 50 women treated in routine clinical practice was 0.5 Gy (range 0.1-2.4) (Table S7c). For the two target combinations available for comparison, oesophagus dose was lower in the audit than in the review of published doses.

References

1. The Royal College of Radiologists. Postoperative radiotherapy for breast cancer: UK consensus statements. The Royal College of Radiologists 2016. https://www.rcr.ac.uk/our-services/all-our-publications/clinical-oncology-publications/postoperative-radiotherapy-for-breast-cancer-uk-consensus-statements/ (accessed December 2023).
2. National Institute for Health and Care Excellence. Early and locally advanced breast cancer: diagnosis and management (NG101). 2023.
3. Feng M, Moran JM, Koelling T, et al. Development and validation of a heart atlas to study cardiac exposure to radiation following treatment for breast cancer. *Int J Radiat Oncol Biol Phys* 2011; **79**(1): 10-8.
4. Kong FM, Ritter T, Quint DJ, et al. Consideration of dose limits for organs at risk of thoracic radiotherapy: atlas for lung, proximal bronchial tree, esophagus, spinal cord, ribs, and brachial plexus. *Int J Radiat Oncol Biol Phys* 2011; **81**(5): 1442-57.

Table S7: Mean whole organ doses from 50 women irradiated for early breast cancer at the Oxford Cancer Centre during 2018 compared with doses from UK breast cancer radiotherapy studies published during 2015-2023

a. Heart

Clinical targets irradiated	Whole heart dose (Gy)					
	Audit			Review*		
	Individual dose estimates (%)	Mean	Range	Individual dose estimates (%)	Average mean	Range
**Left**						
Br/CW only	19 (38)	1.2	0.6-2.6	80 (4)	2.0	1.2-8.0
Br/CW +Ax/SF	11 (22)	1.5	0.8-2.9	1040 (49)	2.7	0.6-5.6
**Subtotal (left)**	**30 (60)**	**1.3**	**0.6-2.9**	**1120 (53)**	**2.6**	**0.6-8.0**
**Right**						
Br/CW only	16 (32)	0.4	0.2-0.8	30 (1)	1.0	0.3-1.0
Br/CW +Ax/SF	3 (6)	0.5	0.3-0.7	983 (46)	1.2	1.0-1.4
Br/CW ±Ax/SF +IM	1 (2)	0.5	–	–	–	–
**Subtotal (right)**	**20 (40)**	**0.4**	**0.2-0.8**	**1013 (47)**	**1.2**	**0.3-1.4**
**TOTAL**	**50**	**1.0**	**0.2-2.9**	**2133**	**1.9**	**0.3-8.0**

b. Lungs

Clinical targets irradiated	Whole lung dose (Gy)					
	Audit			Review*		
	Individual dose estimates (%)	Mean	Range	Individual dose estimates (%)	Average mean	Range
Br/CW only	35 (70)	3.5	2.0-5.8	102 (5)	2.6	1.4-3.0
Br/CW +Ax/SF	14 (28)	4.6	2.8-6.8	2038 (91)	3.0	0.9-5.1
Br/CW ±Ax/SF +IM	1 (2)	4.1	–	90 (4)	7.1	6.7-10.0
**TOTAL**	**50**	**3.8**	**2.0-6.8**	**2230**	**3.1**	**0.9-10.0**

c. Oesophagus

Clinical targets irradiated	Whole oesophagus dose (Gy)					
	Audit			Review*		
	Individual dose estimates (%)	Mean	Range	Individual dose estimates (%)	Average mean	Range
Br/CW only	35 (70)	0.3	0.1-0.4	–	–	–
Br/CW +Ax/SF	14 (28)	1.0	0.5-2.4	36 (47)	1.4	1.0-2.0
Br/CW ±Ax/SF +IM	1 (2)	0.6	–	40 (53)	5.8	1.9-10.0
**TOTAL**	**50**	**0.5**	**0.1-2.4**	**76**	**3.7**	**1.0-10.0**

Footnotes
*Data from review of UK breast cancer radiotherapy studies published during 2015-2023, the results of which are summarized in Figures 1-3. Categories of laterality and targets were included in the table above if there was an equivalent category in the audit. Other categories, e.g. left breast/chest wall±axilla/supraclavicular+internal mammary radiotherapy, were omitted.
Abbreviations: Ax=axilla lymph nodes, Br=breast, CW=chest wall, Gy=gray, IM=internal mammary lymph nodes, RT=radiotherapy, SF=supraclavicular lymph nodes, "–"=unspecified

Text S5: Estimation of absolute mortality radiation risks from typical heart and lung doses in UK breast cancer radiotherapy

Ischaemic heart disease

In the Results section of this systematic review, estimated average mean heart doses from different combinations of laterality and targets irradiated were as follows (Figure 1):

Breast cancer laterality and target	Average mean whole heart dose (Gy)
Left breast/chest wall only:	2.0
Left breast/chest wall + axilla/supraclavicular nodes:	2.7
Left breast/chest wall ± axilla/supraclavicular + internal mammary nodes:	2.9
Right breast/chest wall only:	1.0
Right breast/chest wall + axilla/supraclavicular nodes:	1.2

In the Discussion section of this systematic review, approximate estimates of the 30-year absolute radiation-related risks of death from ischaemic heart disease are presented for typical patients who received breast cancer radiotherapy with the above doses when aged 50 years. These have been reproduced from previously published estimates indicating typical absolute risks of radiation-induced death from ischaemic heart disease by age 80 years for women irradiated at different ages, with and without cardiac risk factors, for different mean heart doses. The relevant section of the table is reproduced overleaf. The estimates assume that the risk of death from radiation-related ischaemic heart disease increases by 7.4% per Gy increase in the mean whole heart dose. They also assume that age-specific mortality rates from ischaemic heart disease and from other causes are equal to those of women in the 15 Western European countries during 2010.

For a woman with no known cardiac risk factors before her radiotherapy, the left-hand panel of the table overleaf was used. For left breast/chest wall radiotherapy, the UK average mean whole heart dose was 2.0 Gy. The corresponding radiation risk from this dose would be 0.3% (red box, left-panel). For a patient *with* known cardiac risk factors before radiotherapy, the right-panel was used and the corresponding radiation-related increase in risk was 0.4% (red box right-panel). For left breast/chest wall + axilla/supraclavicular nodes, 2.7 Gy whole heart dose was the UK average mean dose. This was rounded up to 3 Gy since the table provides estimates from dose in Gy to the nearest whole number. The corresponding radiation risk is represented by the green boxes. It was 0.5% for a woman with no cardiac risk factors prior to radiotherapy and 0.7% for a women *with* prior cardiac risk factors.

This method was used to provide illustrative 30-year risks of radiation-induced heart disease mortality for other average mean heart doses (above) from UK breast cancer radiotherapy to different clinical targets.

Age at irradiation (years)	Mean heart dose (Gy)	No cardiac risk factor: Cumulative risk (%) by attained age				No cardiac risk factor: Absolute risk (%) of radiation-related IHD death by age 80 years	At least one cardiac risk factor: Cumulative risk (%) by attained age				At least one cardiac risk factor: Absolute risk (%) of radiation-related IHD death by age 80 years
		50	60	70	80		50	60	70	80	
50	0		0.09	0.5	1.9	0.0		0.3	0.9	3.4	0.0
	0.5		0.09	0.5	2.0	0.1		0.3	1.0	3.5	0.1
	1		0.09	0.5	2.1	0.2		0.3	1.0	3.6	0.2
	2		0.10	0.5	2.2	0.3		0.3	1.1	3.8	0.4
	3		0.11	0.6	2.4	0.5		0.3	1.1	4.1	0.7
	4		0.11	0.6	2.5	0.6		0.4	1.2	4.3	0.9
	5		0.12	0.6	2.7	0.8		0.4	1.3	4.6	1.2
	6		0.12	0.7	2.8	0.9		0.4	1.3	4.8	1.4
	7		0.13	0.7	2.9	1.0		0.4	1.4	5.0	1.6
	8		0.14	0.7	3.1	1.2		0.4	1.5	5.3	1.9
	9		0.14	0.8	3.2	1.3		0.5	1.5	5.5	2.1
	10		0.15	0.8	3.4	1.5		0.5	1.6	5.8	2.4

From Table S12 in Darby SC, Ewertz M, McGale P, et al. Risk of ischemic heart disease in women after radiotherapy for breast cancer. N Engl J Med 2013; 368(11): 987-98.Copyright (2013) Massachusetts Medical Society. Reprinted with permission,

These illustrative risks are from typical modern UK radiotherapy, and 2010 mortality rates from women in Western Europe. In reality heart doses vary from patient to patient, and mortality rates vary from population to population and from year to year, so an individual woman's absolute risk will vary around these typical values. In addition, there is unavoidable uncertainty in measuring doses in modern radiotherapy planning due to inter- and intra-fraction motion and contouring uncertainties. These estimates do not take into account competing causes of death, so they may overestimate cardiac risk for women at high risk of breast cancer death.

In Darby 2013 cardiac risk factors included:

- Myocardial infarction or coronary revascularisation procedure
- Pericarditis, endocarditis, myocarditis
- Cardiac valve disorders
- Cardiomyopathy
- Cardiac arrhythmia or conduction disorders
- Heart failure

- Other circulatory diseases:

    - Hypertension
    - Cerebrovascular disease
    - Peripheral vascular disease
    - Pulmonary embolism or deep vein thrombosis
- Other:
    - Diabetes
    - Chronic obstructive pulmonary disease
    - Long-term continuous smoking in the year prior to study registration
    - Body Mass Index >30kg/m^2

Reference

Darby SC, Ewertz M, McGale P, et al. Risk of ischemic heart disease in women after radiotherapy for breast cancer. *N Engl J Med* 2013; 368(11): 987-98.

Lung cancer

Illustrative absolute radiation risks from average mean whole lung doses for different combinations of targets irradiated were estimated using the EBCTCG meta-analysis of radiotherapy side-effects [EBCTCG 2017]. In this publication, the rate ratio for incident lung cancer increased by 11% per Gy increase in mean whole lung dose, based on based on data from 40,000 women in 75 randomised trials. In the EBCTCG meta-analysis, this dose-response relationship was combined with baseline annual rates of death from lung cancer for non-smokers in the American Cancer Prevention Study II and for smokers in the UK Million Women study. The estimated absolute 30-year risk of radiation-induced lung cancer death from 5 Gy whole lung dose for a typical non-smoker aged 50 years at irradiation was 0.3%. For a long-term continuing smoker it was 4.4% (see Figure 3A in EBCTCG 2017). The risks of radiation-induced lung cancer mortality in ex-smokers are likely to be much closer to those in never-smokers than in current smokers because stopping smoking substantially reduces lung cancer risk.

These illustrative risks are from typical modern UK radiotherapy and from populations of patients in epidemiological studies. In reality lung doses vary from patient to patient, and mortality rates vary from population to population and year to year, so an individual woman's absolute risk will vary around these typical values. These estimates do not take into account the competing risk of death from breast cancer so the radiation-induced lung cancer risks for patients today may be somewhat lower than this.

In this systematic review, for breast/chest wall only radiotherapy, the average mean whole lung dose was about half of 5 Gy (2.6 Gy). Assuming that the relationship between whole lung dose and radiation-induced lung cancer is linear, the absolute risks from 2.6 Gy would be approximately half the risks from 5 Gy. We estimated absolute risks for different clinical scenarios by multiplying the risks from 5 Gy in the EBCTCG meta-analysis by the fraction of whole lung dose received in each scenario. For example, breast/chest wall irradiation delivered 2.6 Gy lung dose. Therefore the estimated absolute risk in a smoker would be (2.6/5) multiplied by 4.4 = 2.3%.

Study	Clinical scenario	Whole lung dose (Gy)	Absolute 30-year radiation risk (%)	
			Non-smoker	Long term continuing smoker
EBCTCG 2017	Modern breast cancer radiotherapy worldwide 2010-2015	5	0.3	4.4
Current UK review	Breast/chest wall only	2.6	0.2	2.3
	Breast/chest wall + axilla/supraclavicular nodes	3.0	0.2	2.6
	Breast/chest wall ± axilla/supraclavicular + internal mammary nodes	7.1	0.4	6.2

Reference

Early Breast Cancer Trialists' Collaborative Group. Estimating the Risks of Breast Cancer Radiotherapy: Evidence From Modern Radiation Doses to the Lungs and Heart and From Previous Randomized Trials. *J Clin Oncol* 2017; **35**(15): 1641-9.
